# Characteristics of mental health stability during COVID-19: An online survey with people residing in a city region of the North West of England

**DOI:** 10.1371/journal.pone.0266153

**Published:** 2022-07-13

**Authors:** Katalin Ujhelyi Gomez, Rhiannon Corcoran, Adele Ring, Shaima Hassan, Katherine Abba, Jennifer Downing, Mark Goodall, Mark Gabbay, Pam Clarke, Paul Moran, Dorcas Akeju OBE, Kate M. Bennett

**Affiliations:** 1 Department of Primary Care & Mental Health, University of Liverpool, Liverpool, United Kingdom; 2 Public Advisor at the University of Liverpool, Liverpool, United Kingdom; 3 Department of Psychology, University of Liverpool, Liverpool, United Kingdom; Universiti Pertahanan Nasional Malaysia, MALAYSIA

## Abstract

**Background and aim:**

Despite the significant mental health challenges the COVID-19 pandemic and its associated government measures have presented, research has shown that the majority of people have adapted and coped well. The aim of this study was i) to determine the proportion of people with mental stability and volatility during the pandemic in a North West England city region sample and ii) to establish group differences in psychosocial variables. Mental stability and volatility refer to the extent to which individuals reported change in levels of common mental health symptoms over the course of 12 weeks. No change in mental health over the 12 weeks reflected mental stability whilst change in mental health reflected mental volatility.

**Method:**

A two-wave-online survey (N = 163) was used to explore the psychological and social impact of the pandemic on relatively disadvantaged neighbourhoods within the region. The data collected represents 12 weeks of individual pandemic experience between mid-June and mid-December 2020. A three-level composite common mental health change variable was created combining self-reported anxiety and depression to group stable, volatile, and very volatile individuals in terms of the changeability of their mental health. Kruskal-Wallis with post-hoc tests were used to determine how people with mental stability and volatility differed on factors categorised within an ecological framework of resilience (individual, community, societal, and COVID-19 specific).

**Results:**

Individuals categorised as ‘stable’ in terms of mental health symptoms (63.6%) had better mental and physical health; were more tolerant of uncertainty; and reported higher levels of resilience and wellbeing compared to ‘very volatile’ people (19.8%). These individuals also reported feeling less socially isolated, experienced a greater sense of belonging to their community which was more likely to fulfil their needs, and were more likely to have access to green space nearby for their recommended daily exercise. ‘Stable’ individuals did not report worrying any more during the pandemic than usual and tolerated uncertainty better compared to those in the ‘volatile’ group.

**Implications:**

The majority of participants in this sample were mentally stable and coping well with the challenges presented by the pandemic. The resilience of these individuals was related to key place-based factors such as a strong sense of community and useable local assets. The data showcase the role of place-based social determinants in supporting resilience and thereby highlight key preventative measures for public mental health during times of international crisis.

## Introduction

The novel coronavirus SARS-CoV-2, rapidly spread across the world becoming a global pandemic by March 2020. As a response to tackle the virus and limit its spread, the United Kingdom (UK) Government initiated a national lockdown on the 23rd of March 2020 that lasted for over four months. This was followed by various levels of restrictions and public health advice throughout the country for the coming months from social distancing and working from home, mask wearing and hand hygiene through to stringent domestic and international travel restrictions. The ongoing pandemic and the associated government measures have dramatically changed the way people live, work, and socialise [1].

Several research studies have reported that the coronavirus pandemic has presented significant mental health challenges [2–5]. In line with findings in other countries, such as China [6] and Spain [7], the Office of National Statistics (ONS) have reported increased levels of anxiety (32%), and diminished wellbeing (43%), and loneliness (23%) among the UK population [8]. However, some studies have identified different trajectories for mental health [9, 10]. For example, Shevlin et al. (2021) found five classes reflecting stability (low stable and high stable) and improvement, and two classes of deterioration (one more severe than the other). The most common trajectory was a low-stable profile, which could be termed resilient. A systematic review and meta-analysis of 65 pandemic mental health cohort studies suggest that the initial increase in mental health symptoms resolved towards the norm with time, which has been suggested to demonstrate the impact of resilience [11].

### Resilience

**Fig 1:**
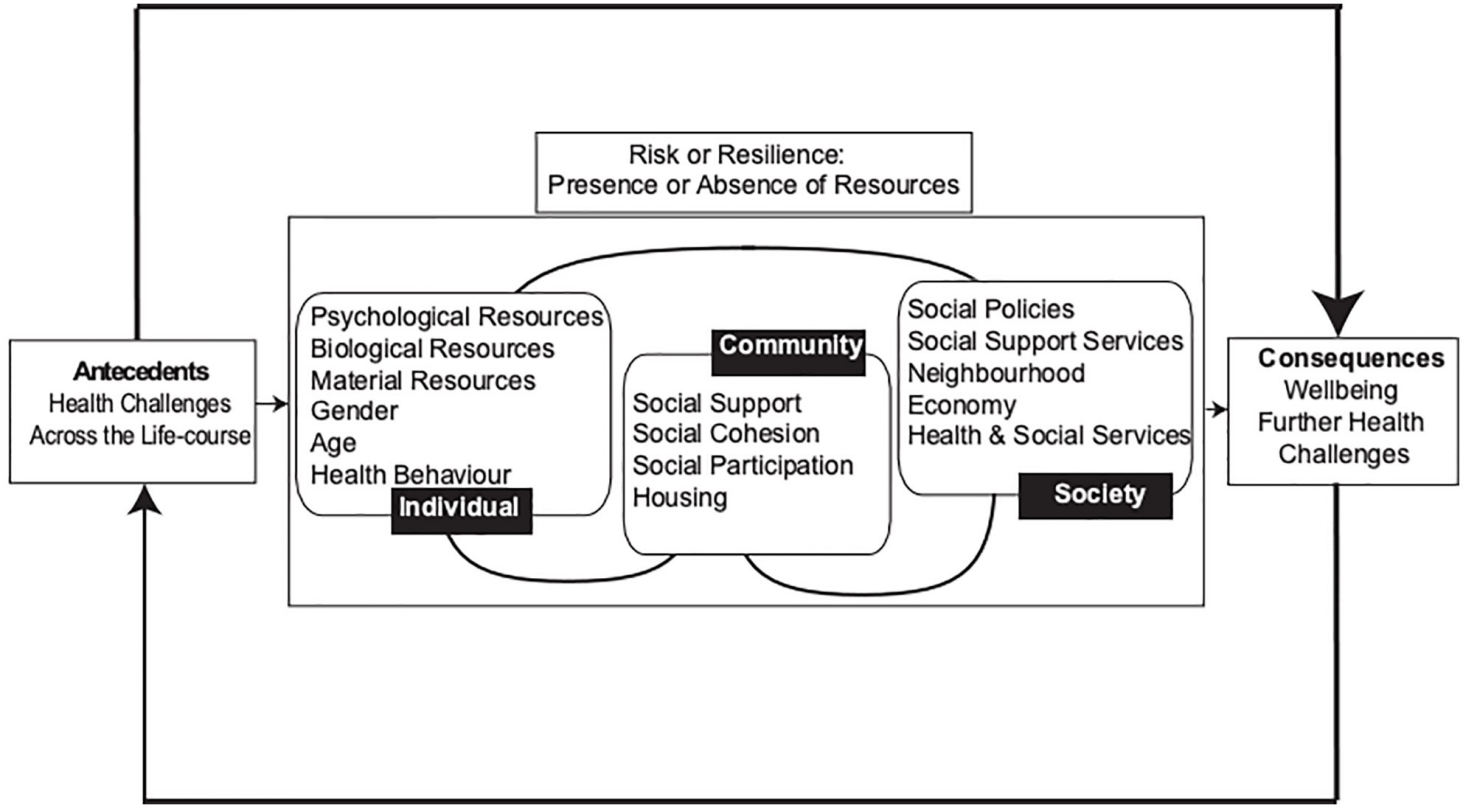
Ecological model of resilience [18].

Resilience is a complex construct that can be understood in different ways. It has been defined as a psychological trait which promotes wellbeing [12] or a process [13] leading to an outcome [14]. Following a comprehensive concept analysis, Windle (2011) [13] defines resilience as “*the process of effectively negotiating*, *adapting to*, *or managing significant sources of stress or trauma*. *Assets and resources within the individual*, *their life and environment facilitate this capacity for adaptation and ‘bouncing back’ in the face of adversity*.*”* [13]. Resilience can also be considered as a capacity for mental stability or toughness [15] through adapting and bouncing back in the face of challenge [14], protecting against stress, trauma, and adversity [16]. However, there are wider determinants of resilient responses to negative situations and trauma [17]. To account for the interaction between different levels of factors that can influence a person’s resilience, an ecological model of resilience has been proposed [18]. This framework (Fig 1) recognises that resilience operates interactively in context at individual, community, and societal levels [13, 19]. Individual resilience thus develops amidst an interplay of environmental-community and social-political factors, most of which are beyond individual control [20].

### Resilience and COVID-19

Higher resilience has been linked to reduced psychological symptomatology [21] and distress [22], as well as increased wellbeing [23–25]. Preliminary data indicates that the pandemic has increased people’s acute and chronic stress levels due to the uncertainty and uncontrollability of the pandemic’s impact on broad aspects of autonomy, health, family and finances [26]. COVID-related resilience has been negatively associated with symptoms of depression, anxiety, somatisation, and negative emotional symptoms [27]. People have been shown to have coped with the pandemic through connections with loved ones, by going outdoors, through physical activity, via spirituality [28, 29], by leading a healthy lifestyle, accepting anxiety and negative emotions, and inquiring information about medical treatment [30]. In a test of the ecological model of resilience during the pandemic in Italy, evidence supporting the role of individual, societal and COVID-specific levels of the model (but not community), was found. Key contributors to resilience in the study included: psychological variables such as conscientiousness and intolerance of uncertainty; demographic variables such as having children in the home and educational level; and COVID-specific variables such as COVID-specific anxiety, and social distancing [31].

In the UK, the COVID-19 crisis has had a particularly negative effect with one of the highest mortality rates in the world [32]. A sense of uncertainty is always challenging and can contribute to the development of generalised anxiety, depression, and health anxiety [33]. A study of 555 UK adults [34] observed high levels of generalised anxiety (27%) as a result of the pandemic that was more than four times the national average pre-pandemic 5.9%; [35]. The figure was also higher compared to rates identified by previous pandemic research (e.g. [36, 37]).

However, increased anxiety, loneliness, distress and low mood are normative experiences during such a crisis and it is one’s response to these negative emotions that ultimately influence longer-term mental health outcomes [38]. Individual responses to stress are affected by coping style, available social support, previous experience with the specific stressor, underlying mental health issues, and personality characteristics [26]. While the negative mental health impact of the pandemic is real, adaptation and recovery are the typical responses to trauma and adversity [39]. This is apparent in research findings among the UK population collected during the pandemic. In spite of struggles as the crisis unfolded in the UK, 64% of people who responded to a national longitudinal survey of 26 016 adults reported coping well [10], using adaptive coping strategies, such as going for a walk, spending time in open spaces, staying connected with others, or exercising [10, 40, 41]. Another study found the prevalence of psychological problems at the early stage of the crisis only slightly higher than during previous pandemics suggesting again the population’s successful adaptation to the situation [42]. As mentioned previously, in a longitudinal study of more than 2000 UK participants conducted during the COVID-19 pandemic, Shevlin et al. (2021) [9] identified five resilience profiles (low stable; high stable; improving; and two deteriorating profiles). Their study found that psychological variables distinguished between the low-stable and the other profiles.

Research that helps us understand the wide range of determinants that impact on individual ability to respond resiliently can facilitate evidence-based policy management of future or ongoing crises. Moreover, while research has been conducted on a national level, in-depth localised research in areas where the pandemic hit harder, as is the case in the cities of the North West of England, is extremely limited.

### The aim of the current study

In order to fill the above research gaps, the current study adopted a resilient systems approach to understand individual responses to the pandemic. Additionally, it investigated the psychological and social impact of the COVID-19 pandemic and its associated lockdown restrictions in a city region located in the North West of England with high levels of disadvantage and thus higher level of vulnerability to the pandemic.

Using Windle and Bennett’s (2011) [18] ecological model of resilience, this study focused on demographic, psychological, community, and societal factors that may determine individual response to the pandemic. Additionally, COVID-19 specific factors were included to assess their capacity to influence resilience. In this study, an individual’s resilience during the pandemic was defined as the extent to which they reported change in levels of common mental health symptoms over the course of 12 weeks. Mental stability may reflect a person’s adaptation and ability to cope with challenges. It is likely that more vulnerable people are more prone to volatility or to change in the experience of mental distress symptoms during challenging times. The study aims to establish i) the proportion of people with mental stability and mental volatility and ii) differences between groups in terms of demographic, psychological, societal, community, and COVID-specific factors.

## Methods

### Design

These data are part of a larger study surveying households early in the COVID-19 pandemic and then twice more within three months. The study explored the psychological and social impact of the pandemic and its associated restrictions on relatively disadvantaged neighbourhoods within a city region in the North West, and factors influencing response to and impact of the pandemic. An online survey using Jisc software was launched in mid-June 2020 and conducted three times, across a 12-week period (week 1, week 6, week 12).

### Participants and recruitment

Participants were engaged using several methods to meet our aim to recruit a representative sample of people living in the disadvantaged communities of a city region in the North West of England. First, the research team re-contacted, by telephone or mail, willing participants who had supplied data for either or both wave 1 or wave 2 of the Household Health Survey conducted in the North West Coast by the Collaborations for Leadership in Applied Health Research funded by the National Institute for Health Research (CLAHRC NWC HHS) [43]. Second, convenience sampling was used advertising the research via local media (newspaper, radio) and social media (Twitter, Facebook). Only people 18 years of age or above residing in the selected city region could participate. The first phase of the survey collected data from 290 residents.

Ethical approval was granted by the University of Liverpool Central Research Ethics Committee (7739). Informed consent was collected at the beginning of the first survey. At the end of each survey, participants were asked for their permission to be contacted for follow-up questions. Non-completion of this survey and follow-up surveys was treated as withdrawal of consent such that only data from individuals who reached the end of the survey was included in this dataset. As a result, missing data was minimal. Through the Participant Information Sheet, participants were reassured that should they experience any distress, they could contact the research team for support. No contacts were made and no support arrangements were requested by any of the participants.

### Data collection and measures

Baseline responses were collected between mid-June and the end of August 2020. While the survey included 3 waves, the data collected at week 6 was a much-abbreviated set of questions focussing on behaviours and pastimes only. The analyses reported here uses the fuller data collected in the first and third waves, representing 12 weeks of individual pandemic experience. The three-wave online survey was first launched on 15/06/2020 and received the first response on 17/06/2020. Recruitment into the first wave of the survey lasted until 31st August 2020. The first response to the week-12 survey was provided on 11/09/2020 and was closed on 04/12/2020. Automated reminders to complete the current wave of the survey were generated through the Jisc software. The research team worked with linked anonymised data.

**Fig 2:**
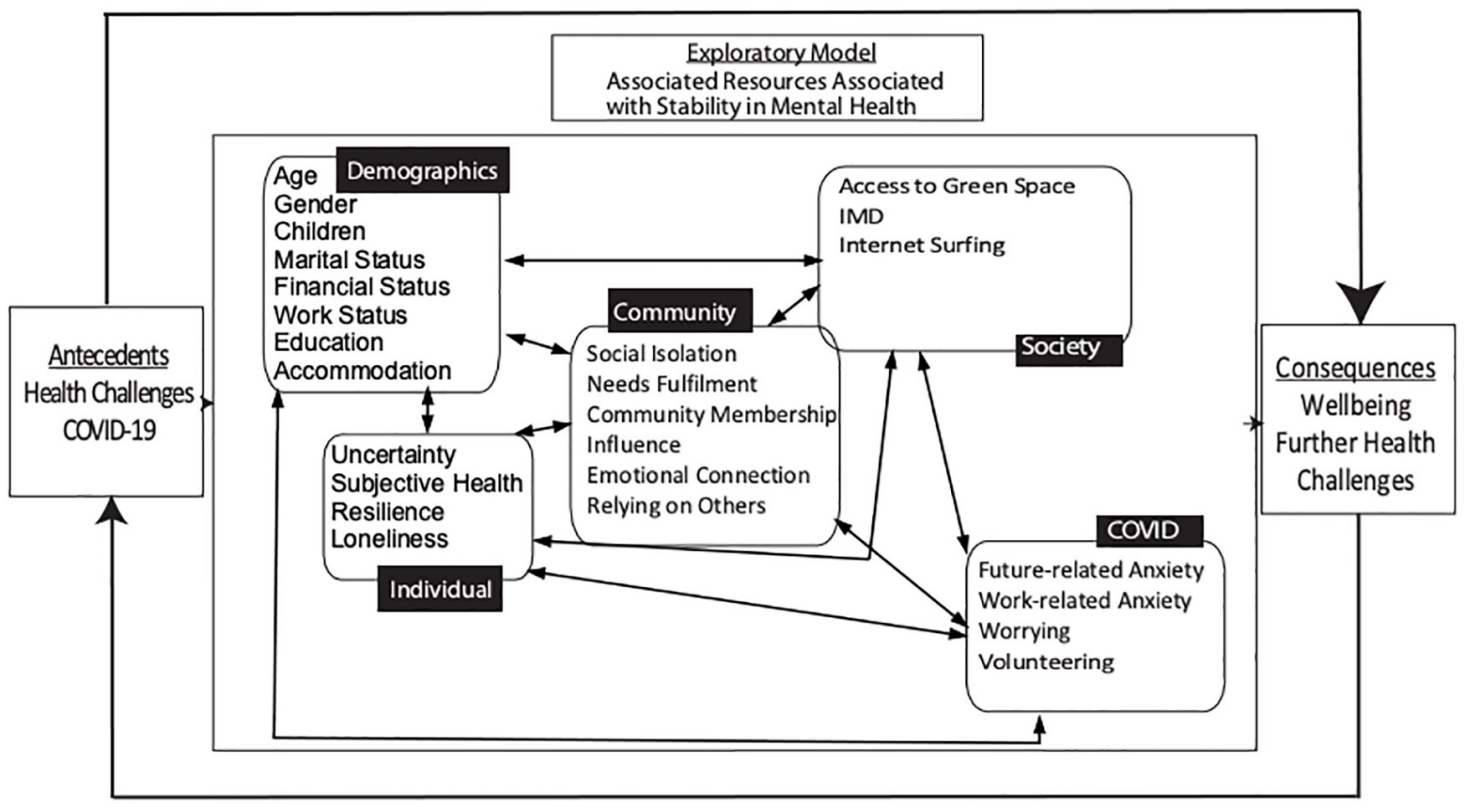
Exploratory model of the associations between the resources in the ecologcial model and mental health.

This extensive survey assessed a wide range of psychological and social determinants. For the full list of measures used refer to S1 Table. This study focuses on a subset of the variables (Fig 2). The first survey comprised multiple measures assessing demographic and socioeconomic factors, mental health and wellbeing, and characteristics at the individual, community, and societal levels. The week-12 follow-up included all questions in the first survey with the exception of the psychological trait measures which would not be expected to change over a 3-months’ time period (e.g. Intolerance of Uncertainty) as these represent steady habitual responses that generally do not alter with changing context. Fig 2 includes the variables used here within the Ecological Model of Resilience.

### Mental health stability outcome variables

The 9-item *Patient Health Questionnaire* (PHQ-9) [44] and the 7-item *Generalised Anxiety Disorder* (GAD 7) questionnaire [45] were used to measure self-reported common mental health symptoms. On both measures, responses range on a four-point Likert scale from 0 ‘not at all’ to 3 ‘nearly every day’. The higher the total score, the more severe the symptoms. In this survey, both scales measured self-reported symptoms taking into consideration how the individual has been feeling over the past week. The PHQ-9 is a valid and reliable measure (Cronbach’s α = 0.86) not only for the identification of depression but also to measure its severity which makes it ideal to track changes in depression levels over time [44]. Similarly, GAD-7 is a valid and efficient tool (Cronbach’s α = 0.91) to screen anxiety and its severity in clinical practice and research [45]. The Cronbach alpha coefficients in the present sample were .90 (PHQ-9) and .91 (GAD-7).

As symptoms of depression and anxiety are typically co-morbid and with a view to meaningfully simplifying subsequent analysis, the GAD-7 and the PHQ-9 scores were summed to create a single common mental health (CMH) variable for the week 1 and week 12 data. The degree of stability or volatility of this CMH variable between week 1 and week 12 provides the working measure of individual resilience through 12 weeks of the pandemic. More detail on this derived variable is presented in the data analysis section below.

### Individual level mechanisms: Demographic and sociodemographic

Demographic characteristics measured included age, gender, marital status, and accommodation. Sociodemographic variables collected were level of education, subjective assessment of financial status before the pandemic; current subjective financial status; work status before the pandemic and current work status.

### Individual level mechanisms: Psychological

The 7-item *Short Warwick–Edinburgh Mental Wellbeing Scale* (SWEMWBS) [46] assesses self-reported subjective psychological wellbeing on a five-point Likert scale over the previous two weeks (Cronbach’s α = 0.91). Responses included 1 = ‘None of the time’, 2 = ‘Rarely’, 3 = ‘Some of the time’, 4 = ‘Often’, 5 = ‘All of the time’. All items are scored positively such that a higher total score represents higher level of wellbeing. The Cronbach alpha coefficient in this sample was .89.

The 6-*item* *Brief Resilience Scale* (BRS) [47] measures self-reported ability to bounce back or to recover from stress (Cronbach’s α = 0.80–0.91). Participants were asked to indicate the extent to which they agreed with each statement on a scale of 1 to 5 where 1 = strongly disagree, 2 = disagree, 3 = neutral, 4 = agree, 5 = strongly agree. Items 1, 3, and 5 of the scale are positively worded. Items 2, 4, and 6 are negatively worded and were reverse coded for scoring. The higher the total score, the more resilient the individual is. The scale has good internal consistency within the sample with a Cronbach alpha of .90.

The BRS was used to test construct validity of the CMH change outcome variable, anticipating that the ‘stable’, ‘volatile’ and ‘very volatile’ groups would differ significantly with respect to their scores on the BRS.

The 12-item two-factor (prospective and inhibitory anxiety) version [48] of the original 27-item Intolerance of Uncertainty Scale (IUS; reported Cronbach’s α = 0.91) [49] was used to quantify emotional, cognitive, and behavioural reactions to ambiguous situations. Items are scored on a five-point Likert scale with response options ranging from 1 = ‘not at all characteristic of me’, 2 = ‘a little characteristic of me’, 3 = ‘somewhat characteristic of me’, 4 = ‘very characteristic of me’, 5 = ‘extremely characteristic of me’. Higher total scores on the scale indicated higher intolerance of uncertainty. In the current study, the Cronbach alpha coefficient was .89.

Participants were also asked whether they had any children, about their health status on a scale between 1 and 100 (where 100 represented the very best of health), if they had felt lonely in the previous week, and about the frequency and type of health service use in the previous 12 weeks.

### Social/ Community mechanisms

*The* *Brief Sense of Community Scale* [50] is an 8-item sense of community scale representing factors of needs fulfilment, group membership, influence and emotional connection to neighbourhood. The scale incorporates a five-point self-report Likert scale with end points from strongly agree to strongly disagree. The measure includes only positively worded items with higher scores indicating stronger sense of community with good internal consistency in the current sample (Cronbach’s α = .91).

Additionally, respondents were asked whether they had access to a pleasant local green/open space for their recommended daily exercise; if they felt isolated from others and, whether they would feel comfortable/uncomfortable asking a neighbour to collect a few shopping essentials for them if necessary. Participants also provided answers regarding whether they surfed the Internet more or less often than usual, or about the same as before the pandemic.

### COVID-19 specific mechanisms

Participants were asked whether they had been or were volunteering to support their local coronavirus action; whether they had experienced worry, and whether they had felt anxious about their own or others’ work situation and / or about the future.

### Data analysis

To facilitate data analyses, some variables were recoded to overcome any limitations posed by low cell counts. See ST 2 for details.

#### The COVID resilience grouping variable

The depression and anxiety total scores on the PHQ-9 and GAD-7 for week 1 (N = 163) and week 12 (N = 162 due to one missing score on the GAD-7) were combined to create composite week 1 and week 12 ‘common mental health’ (CMH) variables (N = 162). A ‘CMH change’ variable was then created (N = 162) to reflect change in reported CMH symptoms over the 12 weeks. This CMH change variable was used to create a stability/volatility variable to capture 3 levels of change to self-reported mental health. Using the sample’s standard deviation of the ‘CMH change’ variable (SD = 8.17), individuals were considered ‘stable’ if their CMH change score fell between -4 and +4. The ‘volatile’ group was made up of people whose CMH change score fell between -4 and -8, or +4 and +8, while individuals whose CMH change score fell anywhere below -8 and above +8 were categorised as ‘very volatile’.

#### Statistical analyses

Due to non-normal distribution of much of the data, non-parametric tests were applied. Kruskal-Wallis tests were used to analyse whether ‘stable’, ‘volatile’, and ‘very volatile’ individuals differed in respect of CMH at week 1 and at week 12 and BRS score at week 1. Follow-up comparisons (Mann-Whitney U tests) used Bonferroni adjustment to control for Type I error associated with conducting multiple tests. Kruskal-Wallis with post hoc tests for continuous variables, and Chi-square test of independence with Fisher’s exact tests for categorical variables were conducted to investigate if ‘stable’, ‘volatile’ and ‘very volatile’ participants differed in terms of their demographic, psychological, community, societal, and COVID-19 specific characteristics. Finally, dependent on variable type, Mann-Whitney U test (continuous) and chi-square test of independence (categorical) compared those who did and did not provide follow-up information at week 12. The analyses were conducted in IBM SPSS Statistics version 26.

## Results

### Characteristics of the sample

Of the 290 participants who completed the first wave, data was available for 163 people at both waves (baseline and 12 weeks’ follow-up). The mean age was 51.5 years ranging between the ages of 22 and 84 years. Sixty-five % of the sample was female and participants were predominantly from white British background (95.7%). Based on changes in self-reported CMH symptoms, the 162 valid cases (due to N = 1 missing anxiety variable at week 12) comprised 103 ‘stable’ (63.6%), 27 ‘volatile’ (16.7%), and 32 ‘very volatile’ (19.8%) individuals. Participant characteristics are reported in Tables 1–3.

**Table 1:** Participant demographic and sociodemographic characteristics.

Demographic and sociodemographic characteristics	N (%)
**Age**	163 (100%)
	Mean age (SD) = 51.50 (14.34)
	Missing 0
**Gender**	
*Male*	47 (28%)
*Female*	106 (65%)
Total	158
Missing	5 (3.68%)
**Ethnicity**	
*Non-white*	6 (3.7%)
*White*	156 (95.7%)
Total	162
Missing	1 (0.61%)
**Marital status**	
*Single/Separated/Divorced/Widowed*	55 (33.7%)
*Married/registered partnership/co-habiting*	103 (63.2%)
Total	158
Missing	5 (3.68%)
**Education**	
*No qualification/GCSE/A level*	46 (28.3%)
*Undergraduate/postgraduate degree*	115 (70.6%)
Total	161
Missing	2 (1.23%)
**Accommodation**	
*House or bungalow*	134 (82.2%)
*Flat/room (Self-contained flat*, *maisonette*, *or apartment/ Room or rooms)*	28 (17.2%)
Total	162
Missing	1 (0.61%)
**Living with others**	
*1 or more people*	120 (73.6%)
*Living alone*	43 (26.4%)
Total	163
Missing	0
**IMD** *	
*≤ 8*.*49*	7 (4.3%)
*8*.*5 to 13*.*79*	35 (21.5%)
*13*.*8 to 21*.*35*	30 (18.4%)
*21*.*36 to 34*.*17*	23 (14.1%)
*≥ 34*.*18*	54 (33.1%)
Total	149
Missing	14 (8.59%)
**Work status before the pandemic**	
*Full-time employed/Self-employed*	82 (50.3%)
*Part-time employed*	30 (18.4%)
*Full-time student/ Part-time student*	6 (3.7%)
*Unemployed/Housewife/housebound*	35 (21.5%)
Total	153
Missing	10 (6.13%)
**Work status currently**	
*Working as normal (Key worker/working in the workplace)*	33 (20.3%)
*Working from home (Employed)*	67 (41.1%)
*Furloughed/unemployed (including unemployed and claiming benefits/not working*?*)*	63 (38.8%)
Total	163
Missing	0

*IMD: Index of Multiple Deprivation quintile group (where ≥ 34.18 is most deprived 20% of Lower-layer Super Output Area, 2019);

**Table 2:** Participant characteristics at week 1 (continuous variables).

	Sample with follow-up data	Total N
	Mean (SD)	
**Individual characteristics**		
Intolerance of uncertainty	32.21(9.56)	163
Subjective health	78.69(17.15)	163
Wellbeing (SWEMWBS)	21.87(4.18)	162
Depression (PHQ-9	5.77(5.87)	163
Anxiety (GAD-&)	4.61(4.97)	163
Resilience (BRS)	3.35(0.84)	163
**Community characteristics**		
Social isolation	2.60(1.32)	163
Sense of community (BSCS)	26.87(6.51)	162

Wellbeing and sense of community missing data: 0.61%.

**Table 3:** Participant characteristics at week 1 (categorical variables).

	Sample with follow-up data N = 163
	N (%)
**Individual characteristics**	
**Anxiety (GAD-7)**	
*Mild*	112 (68.7%)
*Moderate*	30 (18.4%)
*Moderately severe*	16 (9.2%)
*Severe*	6 (3.7%)
*Total*	163
*Missing*	0
**Depression (PHQ-9)**	
*None-minimal*	89 (54.6%)
*Mild*	36 (22.1%)
*Moderate*	25 (15.3%)
*Moderately severe*	7 (4.3%)
*Severe*	6 (3.7%)
*Total*	163
*Missing*	0
**CMH**	
*Stable*	103 (63.6%)
*Volatile*	27 (16.7%)
*Very volatile*	32 (19.8%)
Total	162
Missing	*1 (0.61%)
**Having children**	
*Yes*	37 (22.7%)
*No*	125 (76.7%)
Total	162
Missing	1 (0.61%)
**Finance before**	
*Doing well*	95 (58.3%)
*Getting by/Struggling*	67 (41.1%)
Total	162
Missing	1 (0.61%)
**Finance now**	
*Better off*	52 (31.9%)
*About the same*	88 (54%)
*Worse off*	23 (14.1%)
Total	163
Missing	0
**Loneliness**	
*Never/almost never true*	65 (39.9%
*Occasionally true*	49 (30.1%)
*Sometimes true*	26 (16%)
*Often/almost always/always true*	23 (14.1%)
Total	163
Missing	0
**Community characteristics**	
**Feel comfortable relying on neighbours**	
*Yes*	85 (52.1%)
*No*	78 (47.9%)
Total	163
Missing	0
**BSCS Emotional connection–connected to neighbourhood**	
*Strongly disagree/disagree*	39 (24%)
*Neither agree nor disagree*	39 (24%)
*Agree/strongly agree*	84 (51.5%)
Total	162
Missing	1 (0.61%)
**BSCS Emotional connection–good bond with others in neighbourhood**	
*Strongly disagree/disagree*	34 (20.8%)
*Neither agree nor disagree*	41 (25.2%)
*Agree/strongly agree*	87 (53.4%)
Total	162
Missing	1 (0.61%)
**Societal characteristics**	
**Internet surfing**	
*Less than usual*	2 (1.2%)
*About the same*	97 (59.5%)
*More than usual*	57 (35%)
*Not applicable*	7 (4.3%)
Total	163
Missing	0
**Green space**	
*Yes*	150 (92%)
*No*	13 (8.0%)
Total	163
Missing	0
**Health service use**	
*Yes*	96 (58.9%)
*No*	66 (40.5%)
Total	162 (0.61%)
Missing	1
**Covid-19 specific characteristics**	
**Covid-19 infection** ^1^	
*Yes*, *suspected*	27 (16.6%)
*Yes*, *confirmed*	10 (6.2%)
*No/unknown*	125 (76.7%)
Total	162
Missing	1 (0.61%)
**Worry**	
*Less than usual*	6 (3.7%)
*About the same*	97 (59.5%)
*More than usual*	60 (36.8%)
Total	163
Missing	0
**Work anxiety**	
*Less than usual*	9 (5.5%)
*About the same*	53 (32.5%)
*More than usual*	74 (45.4%)
*I do not work*	25 (15.3%)
Total	161
Missing	2 (1.23%)
**Future anxiety**	
*Less than usual*	11 (6.7%)
*About the same*	88 (54%)
*More than usual*	64 (39.3%)
Total	163
Missing	0
**Volunteering**	
*Yes*	46 (28.2%)
*No*	117 (71.8%)
Total	163
Missing	0

CMH: Change in mental health.

*Missing value is due to N = 1 missing for week-12 anxiety.

^1^Suspected COVID-19 infection: suspected by doctor or oneself. Confirmed COVID-19 infection: confirmed by a positive test or anti-body test.

### Attrition

A total of 127 people was lost to follow-up at week 12 (attrition rate: 56.21%). Mann-Whitney U test showed a significant difference in age between those who completed the week-12 follow-up survey (Md = 52, n = 162) and those who did not (Md = 46, n = 125), U = 8408, z = -2.54, p = .01, r = 0.15, with younger people being more likely to discontinue. People without a degree were less likely to participate in the follow-up survey as demonstrated by a chi-square for independence with Yates Continuity Correction, χ^2^ (1, n = 287) = 13.84, p = .00, phi = .23.

### Stability versus volatility

#### Mental health and resilience

The ‘Stable’, ‘volatile’, and ‘very volatile’ groups differed in respect of mental health at week 1 and week 12. Although there was no significant difference in mental health between the ‘volatile’, and ‘very volatile’ groups, both had significantly worse mental health than their ‘stable’ counterparts at both timepoints. As anticipated, mental stability/volatility was also associated with resilience levels. The mentally stable group scored significantly higher on resilience measured by the BRS compared to those in the ‘very volatile’ group. These findings remained significant at Bonferroni adjusted level (p = .017). Table 4 provides the statistical details.

**Table 4:** Differences between the ‘stable’, ‘volatile’, and ‘very volatile’ groups in terms of mental health and resilience. Significant differences are bolded.

	χ^2^ (p value)	Multiple comparisons	N (%)	Md	Range	Z score	P value (effect size r)
***CMH week 1***	**37.81(0.00)**		162(100)				
		Volatile	27(16.7)	14.00	40.00	-0.83	0.41(0.11)
		Very volatile	32(19.8)	18.50	39.00		
		**Stable**	103(63.6)	4.00	35.00	-5.26	0.00** **(0.45)**
		**Very volatile**	32(19.8)	18.50	39.00		
		**Stable**	103(63.6)	4.00	35.00	-4.26	0.00** **(0.37)**
		**Volatile**	27(16.7)	14.00	40.00		
***CMH follow-up***	**33.48(0.00)**						
		Volatile	27(16.7)	12.00	37.00	-0.36	0.72(0.05)
		Very volatile	32(19.8)	17.00	44.00		
		**Stable**	103(63.6)	5.00	39.00	-4.40	0.00** **(0.38)**
		**Very volatile**	32(19.8)	17.00	44.00		
		**Stable**	103(63.6)	5.00	39.00	-4.67	0.00** **(0.41)**
		**Volatile**	27(16.7)	12.00	37.00		
***BRS Resilience***	**18.64(0.00)**		162(100)				
		Volatile	27(16.7)	3.33	3.33	-1.34	0.18(0.17)
		Very volatile	32(19.8)	2.92	2.83		
		**Stable**	103(63.6)	3.50	3.67	-3.79	0.00** **(0.33)**
		**Very volatile**	32(19.8)	2.92	2.83		
		Stable	103(63.6)	3.50	3.67	-1.51	0.13(0.13)

CMH: Common mental health.

**Significant after Bonferroni adjustment, p<0.017.

Md = Median

#### Demographic factors

No significant differences between the stable, volatile and very volatile groups were identified in terms of demographic factors.

#### Individual factors

The analysis revealed statistically significant differences across ‘stable’, ‘volatile’ and ‘very volatile’ groups for intolerance of uncertainty, subjective health, and wellbeing. Post hoc analyses revealed that the ‘stable’ group reported being significantly more tolerant of uncertainty compared to ‘volatile’ and ‘very volatile’ groups. This group also reported statistically significantly higher levels of subjective health, and wellbeing compared to ‘very volatile’ people.

#### Community factors

There was a difference for self-reported social isolation between the groups with the ‘stable’ group being significantly less likely to be socially isolated than ‘very volatile’ individuals. Significant differences were also found for reported sense of community as measured by the BSCS, for sub-factors of ‘needs fulfilment’ and ‘community membership’. Those with stable mental health over time reported a significantly higher level of needs fulfilment from, and membership of, their community compared to their ‘very volatile’ counterparts. These findings remained significant after Bonferroni adjustment (p < .017).

#### Societal factors

The ‘stable’ group was found to be more likely to report having access to open space than both ‘volatile’ and ‘very volatile’ people. However, after Bonferroni adjustment (p < .017) only the difference between ‘stable’ and ‘very volatile’ groups remained significant.

#### COVID-19-specific factors

There was a statistically significant difference in coronavirus-related worry between the three groups. A significantly higher proportion of the ‘stable’ group reported unchanged levels of worrying compared to the ‘volatile’ and ‘very volatile’ groups with the association between ‘stable’ and ‘volatile’ people remaining significant after Bonferroni adjustment (p < .017).

**Fig 3:**
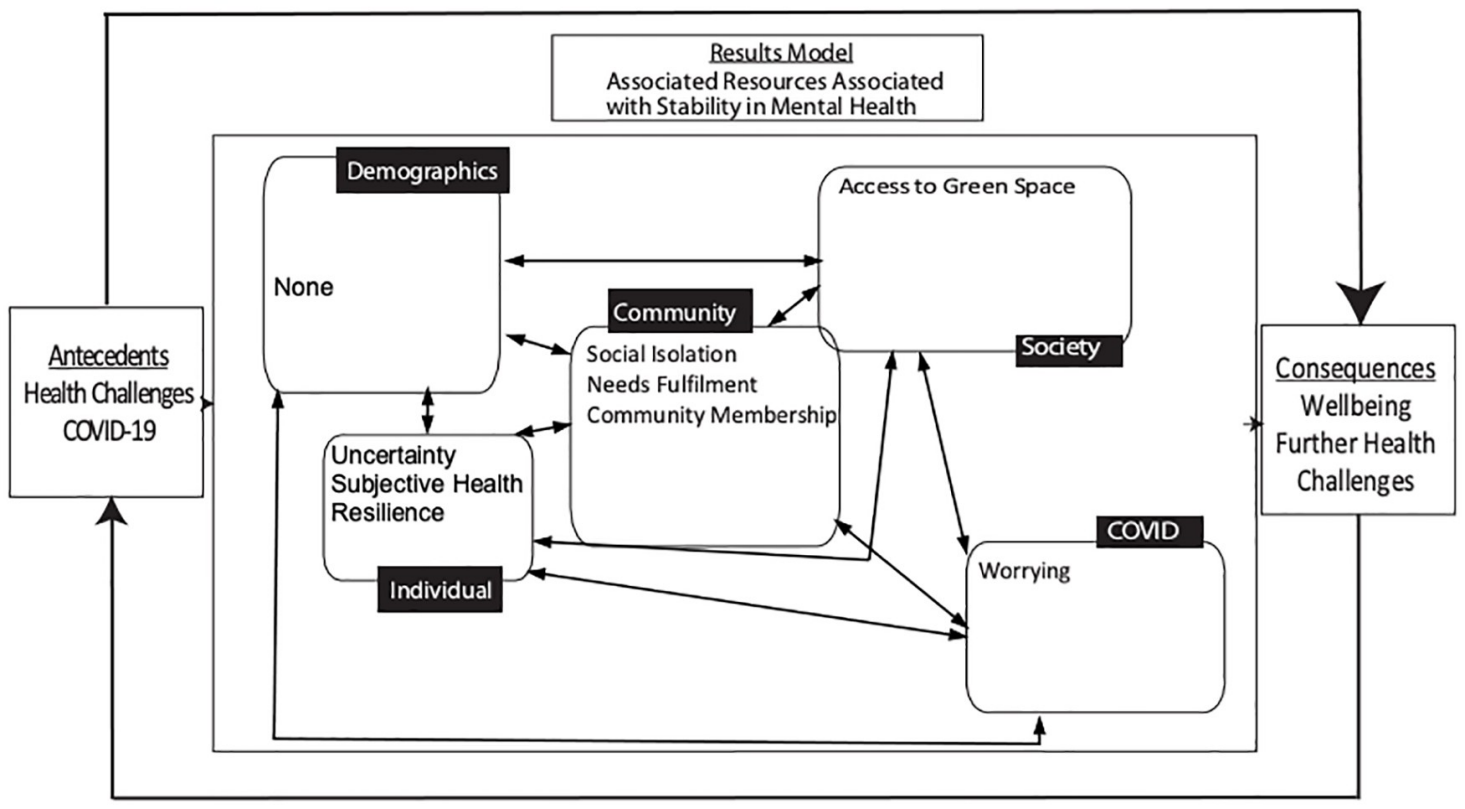
Results model showing the significant associations between resources in the ecological model and mental health.

Fig 3 provides an overview of the results embedded in the resilience framework and Tables 5 and 6 include further information on the data and statistical analyses.

**Table 5:** Differences between the ‘stable’, ‘volatile’, and ‘very volatile’ groups (analysis of continuous variables). Significant differences are bolded.

	χ^2^ (p value)	Multiple comparisons	N (%)	Md	Range	Z score	P value (effect size r)
**Demographics**							
***Age***	2.34(0.310)		162 (100)				
Stable		N/A	103(63.6)	54.00	62.00	N/A	N/A
Volatile		N/A	27(16.7)	50.00	51.00	N/A	N/A
Very volatile		N/A	32(19.8)	52.50	39.00	N/A	N/A
**Individual/psychological**							
***Intolerance of uncertainty***	**14.47(0.001)**		162(100)				
		Volatile	27(16.7)	34.00	35.00	-069	0.493(0.09)
		Very volatile	32(19.8)	38.50	39.00		
		**Stable**	103(63.6)	29.00	35.00	-3.40	0.001** **(0.29)**
		**Very volatile**	32(19.8)	38.50	39.00		
		**Stable**	103(63.6)	29.00	35.00	-2.42	0.016** **(0.21)**
		**Volatile**	27(16.7)	34.00	35.00		
***Loneliness***	5.71(0.06)		162(100)				
Stable		N/A	103(63.6)	2.00	4.00	N/A	N/A
Volatile		N/A	27(16.7)	2.00	4.00	N/A	N/A
Very volatile		N/A	32(19.8)	2.00	4.00	N/A	N/A
***Subjective health***	**12.96(0.002)**		162(100)				
		Volatile	27(16.7)	80.00	70.00	-1.41	0.158(0.18)
		Very volatile	32(19.8)	72.50	78.00		
		**Stable**	103(63.6)	85.00	80.00	-3.41	0.001** **(0.29)**
		**Very volatile**	32(19.8)	72.50	78.00		
		Stable	103(63.6)	85.00	80.00	-1.78	0.75(0.16)
		Volatile	27(16.7)	80.00	70.00		
***Wellbeing (SWEMWBS)***	**16.28(0.00)**		161(100)				
		Volatile	27(16.8)	21.54	18.30	-1.37	0.17(0.18)
		Very volatile	32(19.9)	18.92	16.62		
		**Stable**	102(63.4)	22.78	22.60	-3.93	0.00** **(0.34)**
		**Very volatile**	32(19.9)	18.92	16.62		
		Stable	102(63.4)	22.78	22.60	-1.84	0.066(0.16)
		Volatile	27(16.8)	21.54	18.30		
**Community**							
***Social isolation***	**7.63(0.022)**		162(100)				
		Volatile	27(16.7)	3.00	4.00	-1.19	0.235(0.16)
		Very volatile	32(19.8)	3.00	4.00		
		**Stable**	103(63.6)	2.00	4.00	-2.62	0.009** **(0.23)**
		**Very volatile**	32(19.8)	3.00	4.00		
		Stable	103(63.6)	2.00	4.00	-1.33	0.185(0.12)
		Volatile	27(16.7)	3.00	4.00		
***BSCS–Needs fulfilment***	**7.24(0.027)**		161(100)				
		Volatile	26(16.1)	8.00	6.00	-0.82	0.413(0.11)
		Very volatile	32(19.9)	7.00	8.00		
		**Stable**	103(64)	8.00	8.00	-2.47	0.014** **(0.21)**
		**Very volatile**	32(19.9)	7.00	8.00		
		Stable	103(64)	8.00	8.00	-1.54	.124(0.14)
		Volatile	26(16.1)	8.00	6.00		
***BSCS–Membership***	**6.15(0.046)**		161(100)				
		Volatile	26(16.1)	6.00	8.00	-0.88	0.38(0.12)
		Very volatile	32(19.9)	6.50	8.00		
		Stable	103(64)	8.00	8.00	-1.07	0.29(0.09)
		Very volatile	32(19.9)	6.50	8.00		
		**Stable**	103(64)	8.00	8.00	-2.49	0.013** **(0.22)**
		**Volatile**	26(16.1)	6.00	8.00		
***BSCS–Influence***	0.76(0.68)		161(100)				
Stable		N/A	103(64)	6.00	8.00	N/A	N/A
Volatile		N/A	26(16.1)	5.50	7.00	N/A	N/A
Very volatile		N/A	32(19.9)	6.00	8.00	N/A	N/A
***BSCS–Emotional Connection***	0.76(0.68)		161(100)				
Stable		N/A	103(64)	6.00	8.00	N/A	N/A
Volatile		N/A	26(16.1)	5.50	7.00	N/A	N/A
Very volatile		N/A	32(19.9)	6.00	8.00	N/A	N/A

BSCS: Brief Sense of Community Scale.

**Significant after Bonferroni adjustment, p<0.017.

**Table 6:** Differences between the ‘stable’, ‘volatile’, and very volatile groups analysing categorical variables. Significant differences are bolded.

	Pearson χ^2^ (p value)	Stable N(%)	Volatile N(%)	Very volatile N(%)	Total N(% of total)	Fisher’s exact	Effect size phi
**Demographics**							
***Gender***	1.58(0.45)				152(100)		0.10
Female		67(44.1)	20(13.2)	19(12.5)	106(69.7)	N/A	
Male		31(20.4)	5(3.3)	10(6.6)	46(30.3)	N/A	
***Marital status***	1.75(0.42)				157(100)		0.11
With partner		68(43.3)	17(10.8)	17(10.8)	102(65)	N/A	
Without partner		33(21)	8(5.1)	14(8.9)	55(35)	N/A	
***Education***	1.50(0.47)				160(100)		0.10
Degree or above		75(46.9)	19(11.9)	20(12.5)	114(71.3)	N/A	
Less than degree		27(16.9)	7(4.4)	12(7.5)	46(28.7)	N/A	
***Having children***	0.46(0.79)				161(100)		0.05
Yes		25(15.5)	5(3.1)	7(4.3)	37(23.0)	N/A	
No		77(47.8)	22(13.7)	25(15.5)	124(77.0)	N/A	
***Finance before***	3.42(0.18)				161(100)		0.15
Doing well		65(40.4)	14(8.7)	15(9.3)	94(58.4)	N/A	
Getting by/struggling		37(23.0)	13(8.1)	17(10.6)	67(41.6)	N/A	
***Finance now***	3.10(0.54)				162(100)		0.14
Better off		37(22.8)	8(4.9)	7(4.3)	52(32.1)	N/A	
About the same		54(33.3)	14(8.6)	19(11.7)	87(53.7)	N/A	
Worse off		12(7.4)	5(3.1)	6(3.7)	23(14.2)	N/A	
***Work status before***	11.07(0.09)				153(100)		0.27
Full-time		53(34.6)	13(8.5)	16(10.5)	82(53.6)	N/A	
Part-time		21(13.7)	6(3.9)	3(2.0)	30(19.6)	N/A	
Student		2(1.3)	3(2.0)	1(0.7)	6(3.9)	N/A	
Not working		19(12.4)	4(2.6)	12(7.8)	35(22.9)	N/A	
***Work status currently***	9.10(0.33)				162(100)		0.24
Working in workplace		24(14.8)	6(3.7)	3(1.9)	33(20.4)	N/A	
Furloughed		6(3.7)	2(1.2)	4(2.5)	12(7.4)	N/A	
Working from home		44(27.2)	12(7.4)	11(6.8)	67(41.4)	N/A	
Unemployed		4(2.5)	3(1.9)	3(1.9)	10(6.2)	N/A	
Not working		25(15.4)	4(2.5)	11(6.8)	40(24.7)	N/A	
***Accommodation***	1.59(0.45)				161(100)		0.10
Living in house		88(54.7)	21(13)	24(14.9)	133(82.6)	N/A	
Living in flat		15(9.3)	6(3.7)	7(4.3)	28(17.4)	N/A	
**Individual/psychological**							
***Feel comfortable to rely on others***	3.61(0.16)				162(100)		0.15
Yes		59(36.4)	10(6.2)	16(9.9)	85(52.5)	N/A	
No		44(27.2)	17(10.5)	16(9.9)	77(47.5)	N/A	
**Societal**							
***IMD***	8.11(0.42)				148(100)		0.23
≤8.49		4(2.7)	2(1.4)	1(0.7)	7(4.7)	N/A	
8.5–13.79		27(18.2)	4(2.7)	4(2.7)	35(23.6)	N/A	
13.8–21.35		21(14.2)	2(1.4)	6(4.1)	29(19.6)	N/A	
21.36–34.17		12(8.1)	6(4.1)	5(3.4)	23(15.5)	N/A	
≥34.18		32(21.6)	9(9.1)	13(8.8)	54(36.5)	N/A	
***Internet surfing***	3.68(0.45)				155(100)		0.15
Less than usual		0(0.0)	1(0.6)	1(0.6)	2(1.3)	N/A	
About the same		63(40.6)	15(9.7)	19(12.3)	97(62.6)	N/A	
More than usual		35(22.6)	10(6.5)	11(7.1)	56(36.1)	N/A	
***Green space***	**10.32(0.01)** ^1^				162(100)		0.25
Yes		100(61.7)	N/A	26(16.0)	126(77.8)	**0.01** **	
No		3(1.9)	N/A	6(3.7)	9(5.6)		
**COVID-specific**							
***Volunteering***	1.85(0.40)				162(100)		0.11
Yes		33(20.4)	6(3.7)	7(4.3)	46(28.4)	N/A	
No		70(43.2)	21(13)	25(15.4)	116(71.6)	N/A	
***Worry*** ^2^	**12.65(0.01)**				162(100)		0.28
Less than usual		6(3.7)	0(0.0)	0(0.0)	6(3.7)		
About the same		69(42.6)	12(7.4)	16(9.9)	97(59.9)		
More than usual		28(17.3)	15(9.3)	16(9.9)	59(36.4)		
About the same		69(42.6)	12(7.4)	N/A	81(50.0)	**0.01** **	
More than usual		28(17.3)	15(9.3)	N/A	43(26.5)		
***Future anxiety***	7.00(0.14)				162(100)		0.21
Less than usual		9(5.6)	0(0.0)	2(1.2)	11(6.8)	N/A	
About the same		60(37.0)	14(8.6)	13(8.0)	87(53.7)	N/A	
More than usual		34(21.0)	13(8.0)	17(10.5)	64(39.5)	N/A	
***Work anxiety***	11.00(0.09)				160(100)		0.26
Less than usual		9(5.6)	0(0.0)	0(0.0)	9(5.6)	N/A	
About the same		38(23.8)	8(5.0)	6(3.8)	52(32.5)	N/A	
More than usual		41(25.6)	15(9.4)	18(11.3)	74(46.3)	N/A	

N/A–not applicable; With partner = Married/partnership, Without partner = Single/separated/divorced/widowed; less than degree = GCSE/A level/no qualification; Finance before = self-reported financial situation before pandemic; finance currently = self-reported current financial situation; Full-time = full-time working and self-employment, Part-time = part-time working, Student = full-time and part-time students, Not working = unemployed/housewife/housebound; Working in workplace–including key workers, Unemployed–including those claiming benefits, Not working–e.g. retired; Living in house = living in a house or bungalow or cottage, Living in a flat = living in a self-contained flat/maisonette/apartment or in a room or rooms (e.g. bedsit or flatlet) in a multiple occupancy dwelling or living in a flat in extra care accommodation or other type. Relying on others = feel comfortable/uncomfortable asking a neighbour to collect a few shopping essentials for me if I was ill and no one at home; IMD = Quintile index for multiple deprivation where ≤8.49 is least deprived and ≥34.18 is most deprived 20% of Lower Layer Super Output Areas (2019); Green space = being able to get to pleasant local open or green space for recommended daily exercise; Volunteering = volunteering during pandemic; Worry = worried about things during pandemic; Future anxiety = felt anxious about the future during pandemic; Work anxiety = felt anxious about own work situation or the work situation of others close to the person.

**Significant after Bonferroni adjustment, p<0.017.

^1^No significant differences were found between the stable and volatile groups, and volatile and very volatile groups.

^2^As cell sizes for worrying less during the pandemic than usual were 0, these results were omitted from the table.

## Discussion

The present study aimed to i) determine the proportion of a sample of people living in a city region in the North West of England reporting stable mental health or volatile mental health during a 12-week period of the COVID-19 pandemic and ii) establish whether individual, community, societal, and COVID-19 specific factors were related to stability or change in self-reported symptoms of common mental distress. Participants with low-level or no change in their mental health symptoms over the course of 12 weeks made up the ‘stable’ group, reflecting an ability to adapt and cope well with the challenges of the pandemic. Associations were found between mental health stability/volatility and resilience providing validity for the use of this concept. The findings revealed that nearly two thirds of the participants (63.6%, ‘stable’ group) were coping well during an early 12-week period of the pandemic. Further it was found that individual, community, societal, and COVID-19 specific factors all appeared to contribute to mental stability or volatility.

### Mental stability versus volatility

About one third of the participants were experiencing anxiety and approximately one fourth depression during the first lockdown. This is similar to reports of 32% by the ONS (2020), slightly higher than other previous UK findings of 27% [34], and considerably higher than pre-pandemic figures in the general UK population (5.9%) [35]. However, this heightened level of anxiety is a normal response to an unprecedented public health crisis and lockdown measures. Whether it persists and becomes a long-term mental health problem depends upon a person’s response to the stress and their ability to cope and adjust [38]. The results of this study show that approximately 64% of those who completed the survey at both time points reported a stable level of symptoms of common mental distress (depression and anxiety) over the 12-week period and that for this group, the level of common mental distress reported was significantly lower than for those whose symptoms of common mental distress were more changeable or volatile over time. This is in line with previous research findings [10, 31].

The ecological model of resilience adopted here usefully accounted for the multiple factors, within the individual, as well as their life and environment, that can impact mental health and resilience with group level differences emerging for psychological, community, and societal factors.

At the individual level, mental stability was associated with higher levels of wellbeing and mental and physical health. This link between resilience, coping, mental and physical health, and wellbeing has been clearly indicated by an extensive prior literature [23–25, 27]. Moreover, mental stability during the pandemic was characterised by a better tolerance of uncertainty, which has also been demonstrated in an Italian sample [31]. Previous research has emphasized the relationship between living alone and common mental distress. However, this data did not show any association between change in common mental health and living status. It is possible that the low proportion of people in the sample who lived alone (26.4%) may explain this null finding.

People have been shown to cope with crises through connections with [28] and support from loved ones and others in their community [29] with social connections also affecting how people respond to stress [26]. In our study, mentally stable individuals reported feeling less socially isolated. Community support is important in determining individual resilience [51] and for encouraging healthy coping behaviours [52]. Sense of community, such as feeling a sense of belonging and a perception that one’s needs will be met by the community, has been associated with better mental health [50]. In line with this prior literature, mentally stable people in this study reported higher levels of needs fulfilment and sense of belonging to the community.

At the societal level, those who were coping better reported having an open space nearby which they could use to take the recommended daily exercise during lockdown. There is increasing evidence of the association between wellbeing and outdoor activity and this has also been shown to be the case during the pandemic [28, 29].

COVID-19-related anxiety has been found to hinder resilience [31]. In the present sample, those identified as mentally volatile were characterised by enhanced pandemic-related worry compared to those who were mentally stable and tended to describe their level of worry as ‘about the same’ as prior to the pandemic.

### Implications

These findings demonstrate that mental stability or resilience operates in an interactive way at multiple levels [13, 20]. While individual factors are important in adaptation to adversity and coping with stress, the activation of local assets to enable communities to cope with natural disasters and isolation are also necessary [51]. Therefore, the wider determinants of health operating at the level of community and society, are key to the ability of individuals to cope with crises. With research demonstrating the disproportionate impact of the pandemic on ethnic minority communities [53] and older people [54], the unjust nature of health inequalities operating at societal level are emphasised. These same factors also impact mental ill-health and, importantly, they are factors over which the individual can have very little control. Therefore, public health interventions should focus on developing enabling, supportive environments to facilitate people’s ability to gain control over the determinants of their health and wellbeing [51]. As it seems likely that societal factors also influence people’s propensity to uptake COVID-19 vaccination, future research, should utilise these findings to develop theory and plan future crisis intervention strategies.

### Strengths and limitations

The layered recruitment strategy used in this research ensured the recruitment of a sample from varied neighbourhoods (Table 1) including the most disadvantaged areas of the city region, [55]. Despite the extended recruitment, the week 1 survey sample was relatively small (N = 290) and it was further reduced by high attrition in the 12-week follow-up (N = 163) resulting in very low cell counts in some variables and making interpretation of the results difficult. Additionally, a high proportion of the participants were women, of white ethnic background (although this is representative of the city region demographic), and educated to degree level. These characteristics are common features of online surveys, which reduce the overall representativeness of the sample [56] and in this case, they impacted the ability to focus on the more disadvantaged individuals as originally planned. Further limitation included the sole use of self-report measures, which are prone to desirability bias; and the very low rate of COVID-19 infections among the participants. A sample with a higher infection rate might have coped less well and so presented with more changeable symptoms of CMH. Additionally, those with more profound mental health issues or who experienced other adversities, as well as those who returned to work when restrictions were eased may have been less likely to stay in the study. Finally, the week 1 survey, administered 11 weeks after the nationwide lockdown began, was only able to capture two snapshots of participants’ mental health 12 weeks apart. Defining volatility using only two snapshots is a limitation of this study as the pattern of change in mental health in between time 1 and time 2 and beyond or before snapshots is unknown. Weekly, monthly, or bi-monthly measures over an extended period of time would capture mental health change more reliably. Furthermore, it would be likely to yield data that could be subjected to more sophisticated analytical methods such as path analysis, enabling more to be inferred about the intersectionality of the multi-level influencing factors.

## Conclusion

In line with previous research findings, the majority of the participants (64%) who provided 12-week follow-up data, were mentally stable and coping well with the challenges presented by the pandemic. This is important to record within the context of a relatively disadvantaged urban area. However, it means that the mental health of over a third of the sample during this time of unprecedented adversity was unstable. Understanding the place-based determinants that impact the volatility of mental health is key to facilitate healthy coping and the prevention of mental health problems in the population during a crisis. Attending to the wider determinants of health and developing policies that will help to create places that people can use safely and feel a part of will likely benefit the most vulnerable particularly and in doing so will reduce wellbeing inequity and improve population health.

## Supporting information

S1 Table All measures used in the survey. Measures from which data was used in the current study are bolded.
(DOCX)

S2 Table Recoded variables (N = 290). (DOCX)
