# Correction: Characteristics of mental health stability during COVID-19: An online survey with people residing in a city region of the North West of England

**DOI:** 10.1371/journal.pone.0315612

**Published:** 2024-12-06

**Authors:** The PLOS ONE Staff

The following information is missing from the Funding statement: KUG, RC, AR, SH, KA, JD, M Goodall, M Gabbay, PC, PM, and DA are funded by the National Institute for Health Research (NIHR) Applied Research Collaboration North West Coast (ARC NWC) at the University of Liverpool. The views expressed are those of the author(s) and not necessarily those of the NHS, the NIHR or the Department of Health and Social Care.

The publisher apologizes for the error.
