# Supplementary material for: Characteristics of mental health stability during COVID-19: An online survey with people residing in a city region of the North West of England
Source: PLoS One. 2022 Jul 13;17(7):e0266153. doi: 10.1371/journal.pone.0266153 (PMC9278749; doi:10.1371/journal.pone.0266153)
Supplement: S1 Table — Measures from which data was used in the current study are bolded. (DOCX) [file pone.0266153.s001.docx]

**Supplemental Table 1. All measures used in the survey. Measures from which data was used in the current study are bolded.**

| **Measures** | **Administered at Wave 1** | **Administered at 12-week follow-up** |
| --- | --- | --- |
| **Patient Health Questionnaire (PHQ-9)** | **✓** | **✓** |
| **Generalised Anxiety Disorder (GAD 7)** | **✓** | **✓** |
| **Short Warwick-Edinburgh Mental Well Being Scale (SWEMWBS)** | **✓** | **✓** |
| National Wellbeing / Happiness Measure of the Office for National Statistics (ONS) | ✓ | ✓ |
| **The Brief Resilience Scale (BRS)** | **✓** | **✓** |
| Adult Prosocialness Scale | ✓ | X |
| **Intolerance of Uncertainty Scale - Short version (IUS)** | **✓** | **X** |
| **Brief Sense of Community Scale (BSCS)** | **✓** | **X** |
| **Single-item Relying on neighbours for essentials question** | **✓** | **X** |
| Dispositional/Trait level indicators (Single-item Introversion-Extroversion, Optimism –Pessimism, Sense of Control questions) | ✓ | X |
| **Single-item subjective health status question** | **✓** | **✓** |
| **Health service use (general and COVID-19 related)** | **✓** | **✓** |
| **Single-item volunteering question** | **✓** | **✓** |
| Perception of coronavirus lockdown | ✓ | ✓ |
| The Wellbeing in Place perceptions Scale | ✓ | ✓ |
| COVID-19 knowledge | ✓ | ✓ |
| COVID-19 contact | ✓ | ✓ |
| COVID-19 symptoms | ✓ | ✓ |
| Change in pass times / hobbies (**incl. internet surfing**) | **✓** | **✓** |
| Change in health behaviours / concerns (**incl. worry during the pandemic, future-related anxiety, work-related anxiety**) | **✓** | **✓** |
| **Single-item social isolation question** | **✓** | **✓** |
| **Single-item loneliness question** | **✓** | **✓** |
| Single-item routine change question | ✓ | ✓ |
| **Single-item access to green space question** | **✓** | **X** |
| Pet ownership | ✓ | ✓ |
