# Supplementary material for: Characteristics of mental health stability during COVID-19: An online survey with people residing in a city region of the North West of England
Source: PLoS One. 2022 Jul 13;17(7):e0266153. doi: 10.1371/journal.pone.0266153 (PMC9278749; doi:10.1371/journal.pone.0266153)
Supplement: S2 Table — (DOCX) [file pone.0266153.s002.docx]

**Supplemental Table 2. Recoded variables (N=290).**

| **Variable and original levels** | **N** | **Recoded variable and levels** | **N** |
| --- | --- | --- | --- |
| **Demographic variables** | | | |
| **Ethnicity** |  |  |  |
| *Asian or Asian British* | 8 | *White* | 272 |
| *Black, African, Black British or Caribbean* | 1 | *Non-white* | 16 |
| *Mixed or Multiple Ethnicity* | 5 |  |  |
| *White* | 272 |  |  |
| *Another ethnic background* | 2 |  |  |
| *Prefer not to say* | 2 |  |  |
| Total | 290 | Total | 288 |
| Missing | 0 | Missing (including ‘Prefer not to say’) | 2 |
| **Marital status** |  |  |  |
| *Single* | 76 | *Married/registered partnership/co-habiting* | 171 |
| *Married/registered partnership/co-habiting* | 171 | *Single/Separated/Divorced/Widowed* | 113 |
| *Separated* | 8 |  |  |
| *Divorced* | 15 |  |  |
| *Widowed* | 14 |  |  |
| *Prefer not to say* | 3 |  |  |
| Total | 287 | Total | 284 |
| Missing | 3 | Missing (including ‘Prefer not to say’) | 6 |
| **Education** | | | |
| *No qualification* | 23 | *Undergraduate/postgraduate degree* | 177 |
| *GCSE level or equivalent* | 49 | *No qualification/GCSE/A level* | 110 |
| *A level or equivalent* | 38 |  |  |
| *Undergraduate degree* | 81 |  |  |
| *Postgraduate degree* | 96 |  |  |
| Total | 287 | Total | 287 |
| Missing | 3 | Missing | 3 |
| **Financial status before pandemic** | | | |
| *Doing well* | 148 | *Doing well* | 148 |
| *Getting by* | 125 | *Getting by/Struggling* | 141 |
| *Struggling* | 16 |  |  |
| Total | 289 | Total | 289 |
| Missing | 1 | Missing | 1 |
| **Work status before pandemic** | | | |
| *Full-time employed* | 128 | *Full-time/self-employed* | 147 |
| *Part-time employed* | 48 | *Part-time employed* | 48 |
| *Self-employed* | 19 | *Full-time/part-time student* | 17 |
| *Full-time student* | 15 | *Unemployed/housewife/housebound* | 65 |
| *Part-time student* | 2 |  |  |
| *Housewife/housebound* | 25 |  |  |
| *Unemployed* | 40 |  |  |
| Total | 277 | Total | 277 |
| Missing | 13 | Missing | 13 |
| **Work status currently** | | | |
| *Key worker* | 40 | *Working as normal*  *(Key worker/working in the workplace)* | 68 |
| *Furloughed* | 20 | *Working from home*  *(Employed)* | 105 |
| *Employed and working from home* | 105 | *Furloughed/unemployed*  *(including unemployed and claiming benefits/not working?)* | 117 |
| *Employed and working in the workplace* | 28 |  |  |
| *Unemployed* | 17 |  |  |
| *Unemployed and claiming benefits* | 18 |  |  |
| *Not working (e.g. retired)* | 62 |  |  |
| Total | 290 | Total | 290 |
| Missing | 0 | Missing | 0 |
| **Accommodation** | | | |
| *House or bungalow* | 238 | *House or bungalow* | 239 |
| *Self-contained flat, maisonette, or apartment* | 44 | *Flat/room*  *(Self-contained flat, maisonette, or apartment/* *Room or rooms)* | 49 |
| *Room or rooms in a multiple occupancy dwelling* | 5 |  |  |
| Total | 290 | Total | 288 |
| Missing | 0 | Missing (other type) | 2 |
| **Societal** | | | |
| **Having a garden/yard** |  |  |  |
| *Own garden* | 201 | *Garden or yard space*  *(including shared)* | 270 |
| *Own yard space* | 42 | *No garden or yard space at all* | 20 |
| *Shared garden* | 17 |  |  |
| *Shared yard space* | 10 |  |  |
| *No garden or yard space at all* | 20 |  |  |
| Total | 290 | Total Missing | 290 |
| Missing | 0 |  | 0 |
| **COVID-19 specific** | | | |
| **Volunteering** |  |  |  |
| *I have had no time to volunteer as I work full time.* | 69 | *Volunteering*  *(I have or will volunteer(ed) even though I have concerns for my own or my family/friends health if I do/ I am volunteering to support my local Coronavirus (COVID-19) action.)* | 67 |
| *I would like to volunteer but I am working and do not have time.* | 43 | *No volunteering* | 223 |
| *I do not want to volunteer to support my local Coronavirus (COVID-19) action.* | 26 |  |  |
| *I want to volunteer but cannot because of concerns for my own or my family/friends health if I did.* | 85 |  |  |
| *I have or will volunteer(ed) even though I have concerns for my own or my family/friends health if I do.* | 27 |  |  |
| *I am volunteering to support my local Coronavirus (COVID-19) action.* | 40 |  |  |
| Total | 290 | Total | 290 |
| Missing | 0 | Missing | 0 |
